# Case reports: Central nervous system involvement in patients with newly diagnosed multiple myeloma

**DOI:** 10.3389/fneur.2023.1072490

**Published:** 2023-02-02

**Authors:** Jinghua Liu, Jing Shen, Daihong Liu

**Affiliations:** ^1^Department of Hematology, The Fifth Medical Center of Chinese PLA General Hospital, Beijing, China; ^2^Department of Hematology, Northern Theater General Hospital, Shenyang, China; ^3^Department of Hematology, Capital Medical University Affiliated Beijing Friendship Hospital, Beijing, China

**Keywords:** multiple myeloma (MM), central nervous system, autologous hematopoietic stem cell transplantation, case report, chemotherapy

## Abstract

Multiple myeloma with central nervous system involvement (CNS-MM) is rare, having a poor outcome and occurring in newly diagnosed or relapsed/refractory patients. The current report concerns 3 cases of newly diagnosed MM patients who presented with skull-derived plasmacytomas. Case 1 was a 54-year-old female patient with immunoglobulin D (IgD) subtype who developed extramedullary lesions from the sphenoid and occipital bones and the sphenoid sinus. Cases 2 and 3 had IgA subtype with left or bilateral frontal area lesions. Case 1 was treated with bortezomib, cyclophosphamide and dexamethasone (VCD) as the initial chemotherapy regimen and with bortezomib, lenalidomide, pegylated liposomal doxorubicin and dexamethasone (DVD-R) as the second line regimen. Whole-brain irradiation and intrathecal injection were given but the patient died within 9 months due to disease progression. Case 2 was treated with bortezomib, lenalidomide and dexamethasone (VRD) and received autologous hematopoietic stem cell transplantation (auto-HSCT) with a conditioning regimen of cyclophosphamide, etoposide and melphalan (CEM). Case 3 received DVD-R initially and auto-HSCT with a conditioning regimen of busulfan, cyclophosphamide, and etoposide (BuCyE). Cases 2 and 3 survived until the last follow-up more than 3 years later. Auto-HSCT with modified conditioning regimen as consolidation therapy improved the prognosis of CNS-MM.

## Introduction

Multiple Myeloma (MM) is a malignant proliferation of bone marrow clonal plasma cells (PCs) (1). Extramedullary multiple myeloma (EMM) occurs when a clone and/or subclone of PCs grows outside the bone marrow microenvironment (2). At diagnosis, EMM is typically found in skin and soft tissues; at relapse, typical sites involved include liver, kidneys, lymph nodes, central nervous system (CNS), breast, pleura, and pericardium (3). CNS involvement is rare, occurring in 1% of all MM patients and the prognosis of CNS-MM is very poor with overall survival (OS) reported to be usually < 7 months despite receiving systematic chemotherapy, radiotherapy and lumbar injection (3). The poor prognosis of CNS-MM is not only related to the biological characteristics of the tumor, but also closely related to the fact that most of them occur in several lines of treatment, even after autologous/allogeneic hematopoietic stem cell transplantation (auto/allo-HSCT). The case reports of three patients with CNS involvement at MM diagnosis are presented. Two received auto-HSCT with conditioning regimen of cyclophosphamide, etoposide and melphalan/busulfan, cyclophosphamide, and etoposide (CEM/BuCyE) and survived for more than 3 years. We conclude that auto-HSCT as consolidation therapy with modified conditioning regimen improved the prognosis during treatment of advanced CNS-MM.

## Case presentations

### Case 1

A 54-year-old female patient with an unremarkable medical history presented at our hospital in May 2017 complaining of a 3-week history of dizziness and the absence of the right visual field accompanied by nausea and vomiting. Physical examination showed limited abduction of the right eye and cervical vertebral and cranial computer tomography (CT) was performed in May 2017. CT revealed hyperplasia of the vertebral body and stenosis of the intervertebral space. Cranial magnetic resonance imaging (MRI) showed that multiple spots of slightly longer T1 and T2 signals in bilateral frontal parietal lobe, left temporal lobe subcortical, and bilateral radial crowns, and irregular slightly longer T1 and T2 signals in sellar clivus bone with blurry boundaries. Enhanced scanning showed obvious uneven enhancement. The clivus bone was invaded, which was closely related to the right optic nerve. Whole body positron emission tomography/computer tomography (PET-CT) revealed destruction of the sphenoid and occipital bones and soft tissue masses around the sphenoid and occipital bones and in the sphenoid sinus. The patient was admitted to Northern Theater General Hospital (Shenyang, China) for further investigation and treatment in May 2017.

Laboratory investigations revealed normal levels of hemoglobin, calcium, albumin, globulin, creatinine and serum β2-microglobulin with elevated lactate dehydrogenase (LDH) of 311 U/L (normal range: 109–245 U/L). Serum immunofixation electrophoresis indicated elevated monoclonal immunoglobulin D (IgD) -λ chains. The levels of serum free λ and κ light chains were 132.0 mg/L (normal range: 5.71–26.3) and 6.3 mg/L (normal range: 3.3–19.4), respectively. Bone marrow aspiration revealed 15.5% plasma cells and magnetic bead separation and fluorescence *in situ* hybridization (FISH) showed no deletion of 17p13.1, amplification of 1q21 or fusion of 14q32/16q23, 4p16.3/14q32 or 14q32/20q12. Plasma cells detected in the cerebrospinal fluid (CSF) by flow cytometry were 87.6% positive for CD38 and lambda light chain and negative for CD19.

A diagnosis of CNS-MM was confirmed and bortezomib, cyclophosphamide and dexamethasone (VCD) administered as the initial treatment. However, the patient soon developed a headache and right eyelid ptosis and level of LDH increased to 398U/L. The second-line regimen DVD-R [bortezomib, lenalidomide, pegylated liposomal doxorubicin (PLD) and dexamethasone] was given. The patient's headache abated for 4 weeks before recurring. The patient was given systematic chemotherapy with cisplatin, PLD, etoposide, dexamethasone and lenalidomide (DEDP-R) combined with 30Gy whole-brain irradiation and intrathecal injection of methotrexate and dexamethasone. Following two-cycles of DEDP-R treatment, the patient showed an improvement and plasma cells were negative in the bone marrow as was immunofixation electrophoresis of serum. Two weeks after the end of the last chemotherapy cycle, the patient again developed a headache and died as a result of disease progression. Her OS was only 9 months.

### Case 2

A 61-year-old woman presented at our hospital in February 2019 complaining of a 1-month history of a mass on the left of her forehead. Enhanced cranial CT imaging revealed a space-occupying lesion in the left frontal area and surrounding bone destruction with compression of the brain parenchyma. Then he was admitted to the department of neurosurgery for left frontal mass lesion resection and cranioplasty. Physical examination showed no positive signs besides the left frontal mass. Enhanced MRI revealed a tumor-like lesion of 3.2 × 4.8 cm in the left cranial plate with local bone destruction. Multiple punctate and patchy abnormal signals were observed in the right basal ganglia and bilateral corona radiata and the anterior and posterior horns of both ventricles.

**Figure 1:**
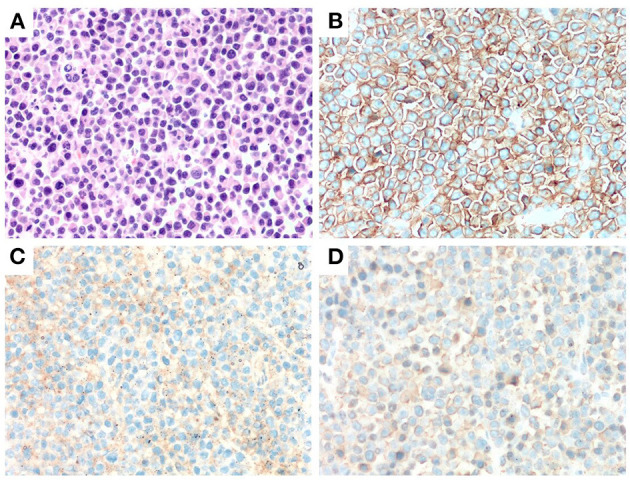
Immunohitsochemical (IHC) analysis of the morphology of left frontal mass in case 2. A markedly proliferation of abnormal plasma cells was observed by H&E staining (× 400) **(A)**. Abnormal plasma cells were positive for CD38 and CD138 (IHC staining × 400) **(B, C)**. Abnormal plasma cells expressed λ (IHC staining × 400) **(D)**.

**Figure 2:**
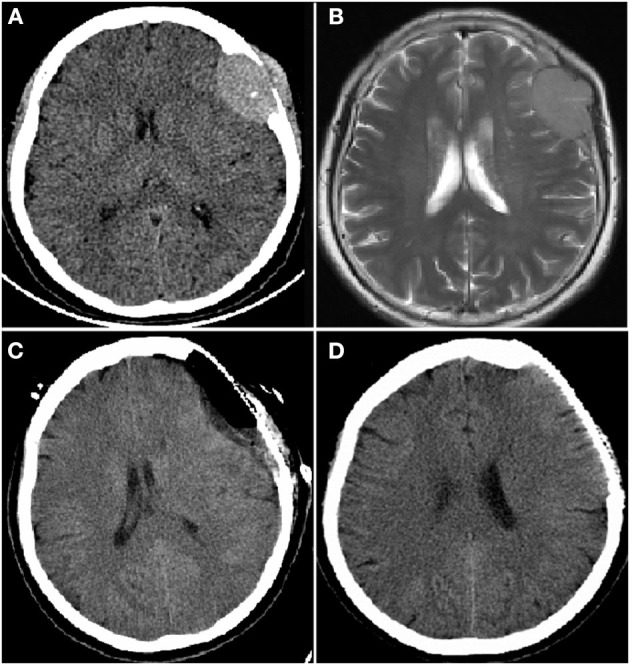
Computer tomography (CT) and magnetic resonance imaging (MRI) brain images from a female patient present with left frontal mass. CT brain image **(A)** and enhanced MRI brain image **(B)** at diagnosis showed a mass of 3.2 × 4.8 cm in left frontal lobe; CT brain image after resection of left frontal lobe mass, the mass disappeared **(C)**; CT brain image before autologous hematopoietic stem cell transplantation (auto-HSCT), no mass in left frontal lobe **(D)**.

Laboratory investigations revealed anemia (white blood cells: 4.3 × 10^9^/L; hemoglobin: 111 g/L; platelets: 135 × 10^9^/L), an increased level of globulin (48.1 g/L) and a decreased level of albumin (39.2 g/L). Mass resection was performed successfully and a pathological examination of the biopsied sample confirmed the presence of extramedullary plasmacytoma. Immunohistochemistry revealed that the tumor cells were positive for CD20, CD38, CD138 and lambda light chain and negative for CD3 and CD21 (Figure 1). Scoring of p53 was about 50%, Ki-67 more than 80% and *in situ* hybridization for EBER was negative. Bone marrow aspiration revealed 35% plasma cells. Magnetic bead separation followed by FISH revealed amplification of 1q21 (92.5%), no deletion of 17p13.1 or fusion of 14q32/16q23, 4p16.3/14q32 or 14q32/20q12. Serum protein electrophoresis (SPE) indicated elevated monoclonal immunoglobulin A (31.35 g/L). The patient received a diagnosis of multiple myeloma IgA λ (DS stage: IIIA; ISS: stage I; ISS-R: stage I) and CNS-MM was confirmed due to skull-derived plasmacytomas. The patient was treated with four cycles of bortezomib, lenalidomide and dexamethasone (VRD) from April 2019. Evaluation by electrophoresis of hematuria immunofixation and flow cytometry of bone marrow aspirate was performed in August 2019 and revealed complete response (CR). The patient was scheduled to receive auto-HSCT. Single-agent cyclophosphamide (3 g/m^2^) was administered in mobilization and peripheral blood stem cells were collected. The patient received auto-HSCT with a cyclophosphamide, etoposide and melphalan (CEM) conditioning regimen in October 2019. Maintenance treatment with single-agent lenalidomide was initiated 3 months after transplantation and the patient remained in CR until October 2022. CT and MRI brain images at each time point are shown in Figure 2.

### Case 3

A 69-year-old woman presented in July 2019 with the complaint of a 3-month history of forehead masses and was admitted to the department of neurosurgery. Enhanced cranial MRI revealed a 5.3 × 7.0 cm round mass in the bilateral frontal region and multiple cranial plate destruction. The patient underwent supratentorial craniotomy and decompressive craniectomy of the bilateral frontal mass lesions. Pathological examination of the biopsied sample confirmed the presence of extramedullary plasmacytoma. Immunohistochemistry revealed tumor cells positive for CD38, CD138, CD56 and lambda light chain and negative for CD3, CD20 and CD21. Ki-67 scoring was 30% and *in situ* hybridization for EBER was negative. The patient discontinued treatment for personal reasons and was re-admitted in an unconscious state in January 2021, and physical examination showed no positive pathological reflex signs.

Laboratory investigations revealed severe anemia and leukopenia (white blood cell: 2.9 × 10^9^/L; hemoglobin: 65 g/L; platelets: 126 × 10^9^/L). Elevated LDH level (407 U/L) and blood calcium (2.9 μmol/L) indicated the scale of the tumor load and elevated creatinine (232.3 μmol/L), increased globulin (63 g/L) and decreased albumin (35.2 g/L) were detected. SPE revealed elevated monoclonal immunoglobulin A(67.67 g/L) and bone marrow aspiration 49% primitive plasma cells. A peripheral blood smear showed mature red blood cells with rouleau-like changes. Whole-body systemic CT showed pathological fracture of the left 3–9 rib and multiple bone lesions in the sternal and T3, 9, 11 and 12 vertebral bones. In addition, pulmonary CT indicated pneumonia and the patient suffered from severe hypoxemia (PO2 53 mmHg).

**Figure 3:**
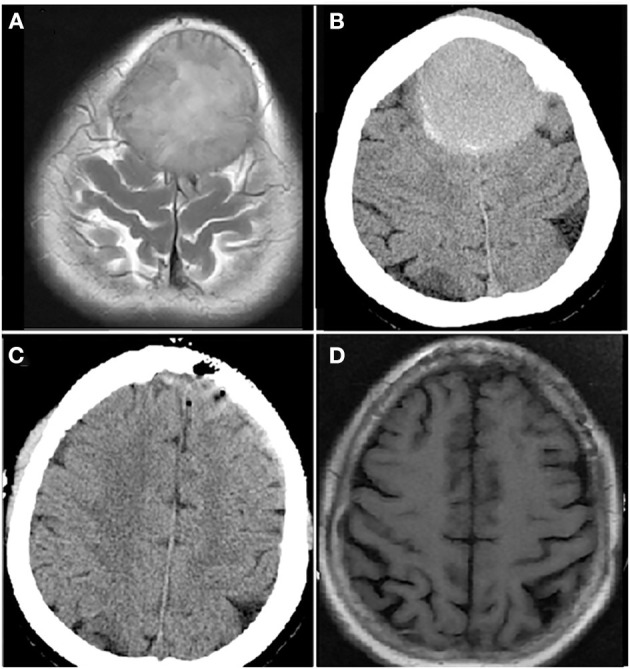
Computer tomography (CT) and magnetic resonance imaging (MRI) brain images from a female patient present with bilateral frontal mass. Enhanced MRI brain image **(A)** and CT brain image **(B)** at diagnosis showed a mass of 5.3 × 7.0 cm in bilateral frontal lobe; CT brain image after resection of bilateral frontal lobe mass, the mass disappeared **(C)**; MRI brain image before autologous hematopoietic stem cell transplantation (auto-HSCT), no mass in bilateral frontal lobe **(D)**.

The patient was diagnosed as CNS-MM IgA λ (DS stage: IIIB; ISS: stage III; ISS-R: stage III) with hypercalcemia, renal insufficiency, pneumonia and hypoxemia. In consideration of infection and decreased renal clearance, VRD with adjusted dose was applied as the initial treatment, but the disease progression was observed in 1 week. The treatment regime was adjusted to DVD-R, which was the addition of PLD on the basis of VRD. By the end of first cycle of DVD-R therapy, the patient's consciousness recovered, creatinine and blood calcium returned to normal level. Three cycles later, the patient can walk slowly. The patient finally achieved CR after 5 cycles of DVD-R therapy, and her performance status returned to normal. Then single-agent cyclophosphamide (3 g/m^2^) was administered in mobilization, peripheral blood stem cells were collected and a cyclophosphamide, etoposide and busulfan (BuCyE) conditioning regimen given in October 2021 for auto-HSCT. Two cycles of VRD were given as consolidation chemotherapy following auto-HSCT. Single-agent lenalidomide was administered for maintenance treatment and the patient remained in CR until October 2022. CT and MRI brain images at each time point are shown in Figure 3.

## Discussion and conclusions

Most cases of CNS involvement in MM have been reported to occur on relapse. A multicenter retrospective study of 172 cases of CNS-MM concluded that 78% patients had disease recurrence (4). The studies of Chen et al. (76%, *n* = 37) and of Gruppo Italiano Malattie EMatologiche dell'Adulto (GIMEMA) (64%, *n* = 50) reached similar conclusions (5). CNS infiltration following auto-HSCT, even allogeneic hemato- poietic stem cell transplantation (allo-HSCT), is not uncommon (6, 7). However, a Brazilian study found that 80% CNS-MM patients (*n* = 20) occurred at diagnosis (8). The current report describes 3 cases of CNS-MM at diagnosis. There have been 7 cases of CNS-MM over the past 10 years at our center, including 4 cases at relapse. This gives a proportion of CNS-MM at diagnosis of 43% (3/7), slightly higher than reports from other centers. Moreover, relatively long OS was also related to the onset time of CNS infiltration at the initial diagnosis.

CNS-MM may be divided into cases with masses, leptomeningeal infiltration or both, according to the results of MRI and CT images and CSF analysis and/or tissue biopsy. Case 1 of the present study had masses and leptomeningeal infiltration while the other 2 cases only had well-demarcated masses. Previous studies have shown significantly better prognosis of patients with simple masses compared with those with leptomeningeal infiltration. These findings were confirmed by the Brazilian study in which OS was not reached for patients with masses lesions as opposed to survival of 5.8 months for those with leptomeningeal infiltration (4, 8). The 2 cases reported here whose survival exceeded 3 years may also have been related to the fact that the lesions did not involve the leptomeninges.

No differences were found in the constituent ratio of immune subtypes between 172 cases of CNS-MM and MM without CNS involvement (4). Cytogenetic analysis of 122 cases showed that 39% (48/122) had del 13q, 23% (28/122) had del 17p, 12% (15/122) had t (4; 14), 7% (9/122) had t (11; 14) and 37% (45/122) had no genetic abnormalities. One of the current 3 cases had the IgD subtype and the other two had the IgA subtype. Cytogenetic analysis was performed for two of the 3 patients, revealing that one had no abnormality and the other had 1q21 amplification.

The permeability of the blood-brain barrier (BBB) to drugs for the treatment of CNS-MM is as important as their efficacy. Proteasome inhibitors (PIs) are the cornerstone of MM therapy but bortezomib has been reported to be ineffective due to its poor CSF uptake (9). The CSF transferability of carfilzomib and ixazomib was also poor and no data is available for their treatment of CNS-MM. Marizomib is a novel irreversible PI that was reported to be effective in 2 patients with refractory CNS-MM (10). Lenalidomide and pomalidomide (POM) are next-generation immunomo- dulatory imide drugs (IMiDs) and both have been shown to have good CNS permeability. Pomalidomide was shown to have higher CNS penetration than lenalidomide (40 vs. 11%) (11) and the combination of lenalidomide and pomadolide have achieved very good efficacy. Lenalidomide has also been used for maintenance treatment (5, 6, 12–14). In addition, PLD is a new doxorubicin dosage form which may be divided into three layers: an inner doxorubicin layer, followed by a phospholipid bilayer and a PEG outer layer. The non-polar, hydrophobic hydrocarbon chain of the phospholipid bilayer greatly enhances the fat solubility of PLD. Perhaps for this reason, PLD inhibits tumor cell growth in the CNS and testes due to its penetration of the BBB and the blood-testis barrier. The current 3 patients received induction therapy of bortezomib, lenalidomide and dexamethasone but disease could only be controlled in case 2. Case 3 achieved CR after the addition of PLD. Despite treatment of case 1 with PLD-based chemotherapy and radiotherapy, the disease could only be controlled in the short term. The question arises of whether any other drugs might have achieved greater success for case 1. Daratumumab, isatuximab and elotuzumab are all antibody-based drugs but there is no data on isatuximab and elotuzumab in CNS-MM. Daratumumab can be detected in the CSF, demonstrating its ability to cross the BBB, and it has been reported to be effective in CNS-MM in combination with intrathecal therapy and/or radiotherapy (15). Selinexor (KPT-330) is a selective nuclear export inhibitor of exportin-1 (CRM1/XPO1) which can cross the BBB, giving favorable CNS penetration. Uptake into the CNS was demonstrated in rats, producing a plasma ratio of 0.72 (16) and a 56% overall response rate (ORR) was shown in 16 patients with EMM (17).

Intrathecal therapy has always been important in the treatment of CNS-MM (4, 15), especially where leptomeningeal involvement is confirmed. Drugs are usually limited to methotrexate and cytarabine which may have no effect on MM, although thiotepa may (18). The current case 1 patient with leptomeningeal involvement confirmed by CSF flow cytometry received intrathecal therapy of methotrexate, cytarabine and dexamethasone. However, the disease still progressed rapidly.

Malignant plasma cells are highly radiosensitive (19) but whole-brain irradiation is of limited utility due to its toxicity. Case 1 was stable for a short time after systematic chemotherapy combined with whole-brain radiotherapy.

Auto-HSCT is the first-line treatment option for newly diagnosed MM. The classic conditioning regimen consists of high-dose melphalan (HD-MEL), while CNS penetration of melphalan is only 10% (20), making HD-MEL a poor choice for a CNS-MM conditioning regimen. The CNS penetrations of busulfan, BCNU, cyclophosphamide and etoposide are >80, 15–70, 20–30, and < 5%, respectively (20). Thiotepa is a cytotoxic alkylating agent, related to nitrogen mustards and has a BBB crossing rate of 100% (21). Investigation of a conditioning regimen of thiotepa and busulfan in newly diagnosed MM achieved longer progression-free survival (PFS) than that of HD-MEL (41.5 vs. 24.4 months) and showed a decreased risk for patients with EMM (HR = 0.43) (22). HD-MEL was not used for our 2 patients who received auto-HSCT. Cases 2 and 3 received a conditioning regimen of CEM and BuCyE and both remained in CR until the last follow-up.

BCMA chimeric antigen receptor T cells (CAR-T) have also shown promising results for CNS-MM, although only a limited number of patients have been treated (14, 23–25). This may prove to be a good option for the treatment of patients with CNS-MM in future. The current treatments for CNS-MM are summarized in Table 1.

**Table 1:** The current treatments for CNS-MM.

**Current treatments**		**CNS permeability**	**Clinical efficacy in CNS-MM**
Proteasome inhibitors	Bortezomib	Poor	Ineffective
	Carfilzomib	Poor	No data
	Ixazomib	Poor	No data
	Marizomib	Good	Effective in case reports
Immunomodulatoryimide drugs	Lenalidomide	40%	Effective
	Pomadolide	11%	Effective
Anthracyclines	Polyethylene glycol liposome doxorubicin	No data	Effective in case reports
Monoclonal antibodies	Daratumumab	Good	Effective
	Isatuximab	No data	No data
	Elotuzumab	No data	No data
Selective nuclear export inhibitors	Selinexor	Good	Effective
Intrathecal therapy		Effective for leptomeningeal infiltration	
Whole-brain irradiation		Effective but limitation due to its toxicity	
Autologous hematopoietic stem cell transplantation		Optimal conditioning regimen needs to be explored	
BCMA CRA T cells therapy		Effective	

The diagnosis and treatment of 3 cases of CNS-MM were presented in our study, but the potential limitations in treatment was inevitable. For example, VCD as the initial chemotherapy regimen for case 1, which may miss opportunity for other effective drugs, such as pomadomide, daratumumab, selinexor and thiotepa, since some drugs may not be available at that time. Moreover, conditioning regimen containing thiotepa and busulfan may improve the prognosis based on recent reports, although case 2 with CEM conditioning regimen achieved over 3-year remission.

## Data availability statement

The original contributions presented in the study are included in the article/supplementary material, further inquiries can be directed to the corresponding authors.

## Ethics statement

The studies involving human participants were reviewed and approved by the Ethics Committee of Northern Theater General Hospital. The patients/participants provided their written informed consent to participate in this study. Written informed consent was obtained from the individual(s) for the publication of any potentially identifiable images or data included in this article.

## Author contributions

JL and JS: concept, design, and draft the manuscript. DL: concept and design. All authors contributed to the article and approved the submitted version.
